# Effect of autologous hematopoietic stem cell transplantation for patients with peripheral T-cell lymphoma in China: A propensity score-matched analysis

**DOI:** 10.3389/fonc.2022.1039888

**Published:** 2022-11-17

**Authors:** Hongye Gao, Meng Wu, Shaoxuan Hu, Ning Ding, Xinqiang Ji, Lan Mi, Xiaopei Wang, Yuqin Song, Jun Zhu, Weiping Liu

**Affiliations:** ^1^ Key Laboratory of Carcinogenesis and Translational Research (Ministry of Education), Department of Lymphoma, Peking University Cancer Hospital & Institute, Beijing, China; ^2^ Key Laboratory of Carcinogenesis and Translational Research (Ministry of Education), Department of Medical Record Statistics, Peking University Cancer Hospital & Institute, Beijing, China

**Keywords:** peripheral T-cell lymphoma, survival, autologous hematopoietic stem cell transplantation (autoHCT), outcomes - health care, propensity score matching (PSM)

## Abstract

**Background:**

The role of consolidation therapy with autologous stem cell transplantation (ASCT) in patients with peripheral T-cell lymphoma (PTCL) in first complete remission (CR1) or partial remission (PR1) remains controversial. The existing data from China are limited. Therefore, we aimed to investigate the effect of ASCT on the survival of Chinese patients with PTCL showing response to induction chemotherapy at our hospital.

**Methods:**

We retrospectively reviewed the data of patients with PTCL (excluding Natural killer/T cell lymphoma) in CR1 or PR1 treated at Peking University Hospital &Institute from 1996 to 2020. Propensity score matching (PSM) was used to balance clinical characteristics between the ASCT and non-ASCT groups. The primary endpoints were event-free survival (EFS) and overall survival (OS).

**Results:**

Of the 414 selected patients, 73 received ASCT consolidation and 341 did not. Over a median follow-up of 5.7 years, survival was significantly better in the ASCT group than in the non-ASCT group (median EFS, 8.1 years vs. 2.8 years, *P* = 0.002; median OS, 14.9 years vs. 10.2 years, *P* = 0.007). The 5-year EFS and OS rates were 68.4% and 77.0% in ASCT group, and 43.2% and 57.6% in non-ASCT group, respectively. The survival benefit was confirmed in the propensity score matched cohort (46 patients who received ASCT and 84 patients who did not receive ASCT): *P* = 0.007 for median EFS and *P* = 0.022 for the median OS. Cox regression analysis showed that ASCT was independently associated with better survival: hazard ratio (HR) for EFS, 0.46 (95% CI: 0.28-0.76); HR for OS, 0.50 (95% CI: 0.31-0.84). Subgroup analysis showed that ASCT was more likely to benefit higher-risk patients and those with advanced disease. Among the subtypes of PTCL, the benefit was significant in angioimmunoblastic T-cell lymphoma (HR = 0.26 [95% CI: 0.10-0.66] for EFS and 0.29 [95% CI: 0.12-0.74] for OS), but not in the other subtypes.

**Conclusion:**

ASCT may improve the long-term survival of patients with PTCL in first CR or PR, especially for patients with angioimmunoblastic T-cell lymphoma. The specific groups most likely to benefit from upfront ASCT need to be clearly identified.

## Introduction

Peripheral T/NK-cell lymphoma (PTCL) is a group of rare and highly heterogeneous malignant lymphoproliferative diseases originating from mature T- and NK-cells. PTCL is classified into subtypes such as angioimmunoblastic T-cell lymphoma (AITL), ALK-positive anaplastic large-cell lymphoma (ALK+ALCL), ALK-negative anaplastic large-cell lymphoma (ALK-ALCL), peripheral T-cell lymphoma not otherwise specified (PTCL-NOS), and so on (1, 2). Most subtypes have a dismal prognosis (3, 4). Due to the rarity and heterogeneity of PTCL, there is still no consensus on the best treatment approach. The role of autologous stem cell transplant (ASCT) consolidation continues to be debated (5–10).

Current guidelines recommended ASCT for most PTCL patients achieving remission after induction chemotherapy (11–13). The National Comprehensive Cancer Network and the American Society for Transplantation and Cellular Therapy recommend ASCT for patients with first-time complete remission (CR1) (11, 12). Meanwhile, the European Society of Medical Oncology guidelines suggests that patients with first-time partial remission (PR1) could also be considered for ASCT (13). However, these recommendations were based on low-quality evidence from retrospective studies (14–17) and a few single-arm prospective studies (5, 18–20). There remains no consensus on the utility of consolidative ASCT.

One real-world study of lymphoma in a Swedish population showed that PTCL patients (excluding those with ALK+ALCL) receiving ASCT had better progression-free survival (HR: 0.56 [0.39-0.80]) and overall survival (OS; HR: 0.58 [0.40-0.84]), but the control group may enroll patients who did not respond to the initial chemotherapy (17). Comprehensive Oncology Measures for Peripheral T-Cell Lymphoma Treatment (COMPLETE) study showed a statistically nonsignificant tendency for better survival following ASCT in PTCL with CR1: the median OS in patients who underwent ASCT was not reached, versus 57.6 months in patients who did not receive ASCT (*P* = 0.06) (21). The large Lymphoma Study Association (LYSA) Registry-based multicenter retrospective study investigated the effect of ASCT in 134 patients with CR1 or PR1. Neither propensity score-matched analysis (*P* = 0.90 for PFS, *P* = 0.66 for OS, respectively) nor Cox multivariate analysis (HR = 1.02 [95% CI: 0.69-1.50] for PFS; HR = 1.08 [95% CI:0.68-1.69] for OS) showed a survival advantage with upfront ASCT consolidation (22).

To the best of our knowledge, no well-designed randomized controlled trial has been conducted so far to evaluate the impact of ASCT in the clinical setting. The data based on Chinese patient population is limited. Therefore, in the present study, we aimed to investigate the effect of ASCT on the survival of patients with PTCL showing response to induction therapy.

## Methods

### Patients

The study was granted by the ethics committee of Peking University Cancer Hospital & Institute. Informed consent was exempted because the data were analyzed anonymously. A total of 8,826 patients with lymphoma were admitted to Peking University Cancer Hospital & Institute from January 1996 to November 2020. Pathological diagnosis was based on the corresponding criteria (1, 23–25). Patients with mature T-cell lymphoma who achieved CR1/PR1 were included in this study. The main exclusion criteria included patients with natural killer/T cell lymphoma (NK/TCL), receiving allogeneic transplantation, and missing clinical data. Assessment of response to initial treatment was evaluated using the Lugano criteria (26), and ^18^F-FDG positron emission tomography/computed tomography (PET/CT) was introduced since 2009. A total of 414 patients met the eligibility criteria. **Supplementary Figure 1** shows the patient selection process. The selected patients were classified into risk groups using the International Prognostic Index (IPI) (27).

### Definitions

Event-free survival (EFS) was defined as the time from the initial diagnosis to the date of occurrence of disease recurrence/progression confirmed by clinical manifestations, imaging or pathological reports, initiation of second-line salvage therapy, or death (whichever occurred first). OS was defined as the time from the initial diagnosis to death due to any cause. None of the above events that occurred at the last follow-up of the patient were censored. Follow-up data were collected *via* telephone interviews and a review of inpatient and outpatient medical records.

### Statistical analysis

Statistical analysis was done using R 4.2.0 (https://www.r-project.org). Categorical variables were summarized as percentages and tested by the chi-square test or Fisher exact test. Propensity score matching (PSM) was performed to compare EFS and OS between ASCT and non-ASCT groups after balancing clinical parameters, which were selected according to previous literature and clinical experience; the parameters were histological subtype, age, sex, lactate dehydrogenase level (LDH), Ann Abor stage, Eastern Cooperative Oncology Group (ECOG) performance status, B symptoms, and response to the initial treatment. The caliper value was set to 0.25, and the matching ratio was 1: 2 (ASCT: non-ASCT). PSM was conducted using the “MatchIt” package in R. Survival analysis was performed before and after matching, using Kaplan–Meier curves and tested by the log-rank test. Univariate and multivariate Cox regression analyses were conducted to determine the independent effect of ASCT on survival after adjusting for other variables (sex, age, Ann Abor stage, IPI risk group, and histological subtype [ALK+ALCL *vs.* non-ALK+ALCL]). All significant factors from the univariate analysis were included in the multivariate analysis. Survival rates were estimated by the Cox proportional hazards model, Hazard ratios, with 95% confidence intervals, were calculated to quantify risk. Two-sided *P* < 0.05 was considered statistically significant.

## Results

### Patient characteristics

Of the 414 selected patients, 73 received ASCT (ASCT group) and 341 did not receive ASCT (non-ASCT group). As shown in **Table 1**, the histologic subtypes were ALK+ALCL (n = 85, 20.5%) AITL (n = 116, 28.0%), ALK-ALCL (n = 42, 10.1%), PTCL-NOS (n = 76,18.4%), and others (n = 95, 22.9%). The first-line regimens were mainly CHOP (cyclophosphamide, doxorubicin, vincristine, and prednisolone) and CHOPE (CHOP plus etoposide). The details are available in **Supplemental Table 1** and **Table 2**. In the ASCT group, the bloodstream infection, febrile neutropenia, and 100-day treatment related mortality rates were 4.4%, 71.1% and 0, respectively.

**Table 1:** Baseline characteristics of patients in the ASCT group and the non-ASCT group.

	Before matching			After matching		
	Non-ASCT	ASCT	*P*	Non-ASCT	ASCT	*P*
	n = 341	n = 73		n = 84	n = 46	
**Subtypes (%)**			0.540			0.860
ALK+ALCL	67 (19.6)	18 (24.7)		19 (22.6)	12 (26.1)	
ALK-ALCL	35 (10.3)	7 (9.6)		8 (9.5)	4 (8.7)	
AITL	92 (27.0)	24 (32.9)		32 (38.1)	17 (37.0)	
PTCL-NOS	66 (19.4)	10 (13.7)		4 (4.8)	4 (8.7)	
Others*	81 (23.8)	14 (19.2)		21 (25.0)	9 (19.6)	
**Age, years, median (IQR)**	53.0 (35.0, 64.0)	42.00 (26.0, 51.0)	< 0.001	44.0 (25.0, 59.0)	43.00 (29.0, 54.0)	0.536
**Female (%)**	110 (32.3)	21 (28.8)	0.657	25 (29.8)	11 (23.9)	0.612
**LDH ≥ULN (%)**	102 (34.5)	33 (61.1)	< 0.001	42 (50.0)	26 (56.5)	0.597
**III–IV stage (%)**	226 (66.3)	61 (83.6)	0.006	70 (83.3)	39 (84.8)	> 0.99
**ECOG ≥2 (%)**	23 (7.2)	5 (8.2)	0.993	7 (8.3)	4 (8.7)	> 0.99
**B symptoms (%)**	153 (45.1)	42 (57.5)	0.073	48 (57.1)	28 (60.9)	0.821
**PR to first treatment (%)**	121 (35.5)	23 (31.5)	0.609	25 (29.8)	14 (30.4)	> 0.99

AITL, angioimmunoblastic T-cell lymphoma; ALK-ALCL, anaplastic large-cell lymphoma, anaplastic lymphoma kinase negative; ALK+ALCL, anaplastic large-cell lymphoma anaplastic lymphoma kinase positive; PTCL-NOS, PTCL not otherwise specified; LDH, lactate dehydrogenase; ASCT, autologous stem cell transplantation; PR, partial remission; ULN, upper limit of normal; ECOG, Eastern Cooperative Oncology Group performance status.

* Others indicates patients with primary cutaneous anaplastic large cell lymphoma, mycosis fungoides/sézary syndrome, hepatosplenic T-cell lymphoma, enteropathy associated T cell lymphoma, subcutaneous panniculitis-like T-cell lymphoma, monomorphic epitheliotropic intestinal T-cell lymphomas, lymphomatoid papulosis, primary cutaneous CD8+ aggressive epidermotropic cytotoxic T-cell lymphoma and other rare subtypes. A portion of patients were classified in this group due to the limitation of the diagnostic measurements in the early era (e.g ALCL with uncertain ALK status).

### Survival analysis in unmatched data

**Figure 1:**
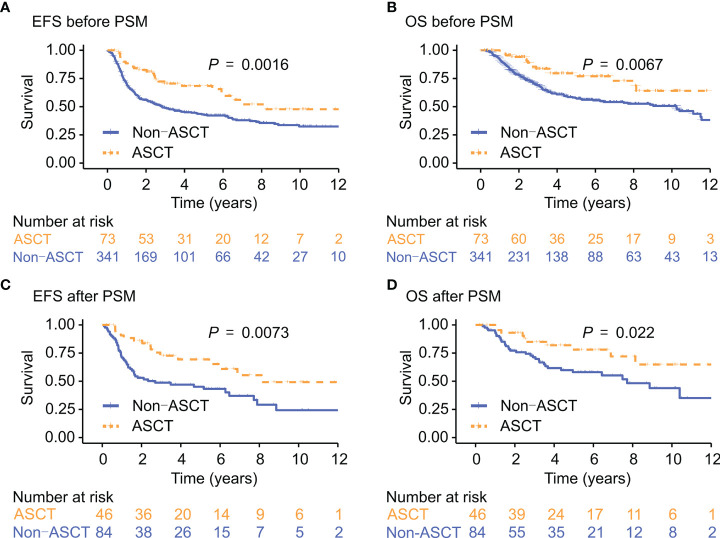
EFS and OS in the ASCT group and the non-ASCT group before and after PSM. EFS, event-free survival; OS, overall survival; ASCT, autologous stem cell transplantation; PSM, propensity score matching. Kaplan-Meir analysis of EFS **(A, C)** and OS **(B, D)**, before and after PSM.

The median follow-up was for 5.7 years. Median EFS and OS were significantly better in the ASCT group than in the non-ASCT group (EFS: 8.1 years vs. 2.8 years, *P* = 0.002; OS: 14.9 years vs. 10.2 years, *P* = 0.007). The 5-year EFS and OS rates were 68.4% (95% CI: 57.7%-81.1%) and 77.0% (95% CI: 66.5%-89.1%), respectively, in the ASCT group vs. 43.2% (95% CI: 37.8%-49.3%) and 57.6% (95% CI: 52.0%-63.8%), respectively, in the non-ASCT group (*P* < 0.001 for all; **Figure 1**).

As expected, patients with ALK+ALCL had better survival outcomes (**Supplementary Figures 2A**, **2B**; **Supplementary Table 3**). We also compared the survival outcome between patients with CR and PR in **Supplementary Figures 3A, B**. Patients with CR had better 5-year EFS (74.2% vs. 53.6%) and OS (85.0% vs. 69.0%) than those with PR (57.1% vs. 23.8%; 59.5% vs. 37.2%) in the ASCT and non-ASCT group, respectively (**Supplementary Figures 3C–F**).

### Survival analysis in matched data

In the propensity score matched (PSM) cohort, there was no statistically significant differences in clinical characteristics between patients in the ASCT group (n = 46) and the non-ASCT group (n = 84; **Table 1**). In this cohort, median EFS and OS were also better in the ASCT group than in the non-ASCT group (EFS: 8.1 years vs. 2.7 years, *P* = 0.007; OS: “not reached” vs. 7.7 years, *P* = 0.022). In addition, the survival difference remained statistically significant when excluding patients with ALK+ALCL after performing PSM method (**Supplementary Figures 2C**, **2D**; **Supplementary Table 4**).

### Cox regression analysis

**Figure 2:**
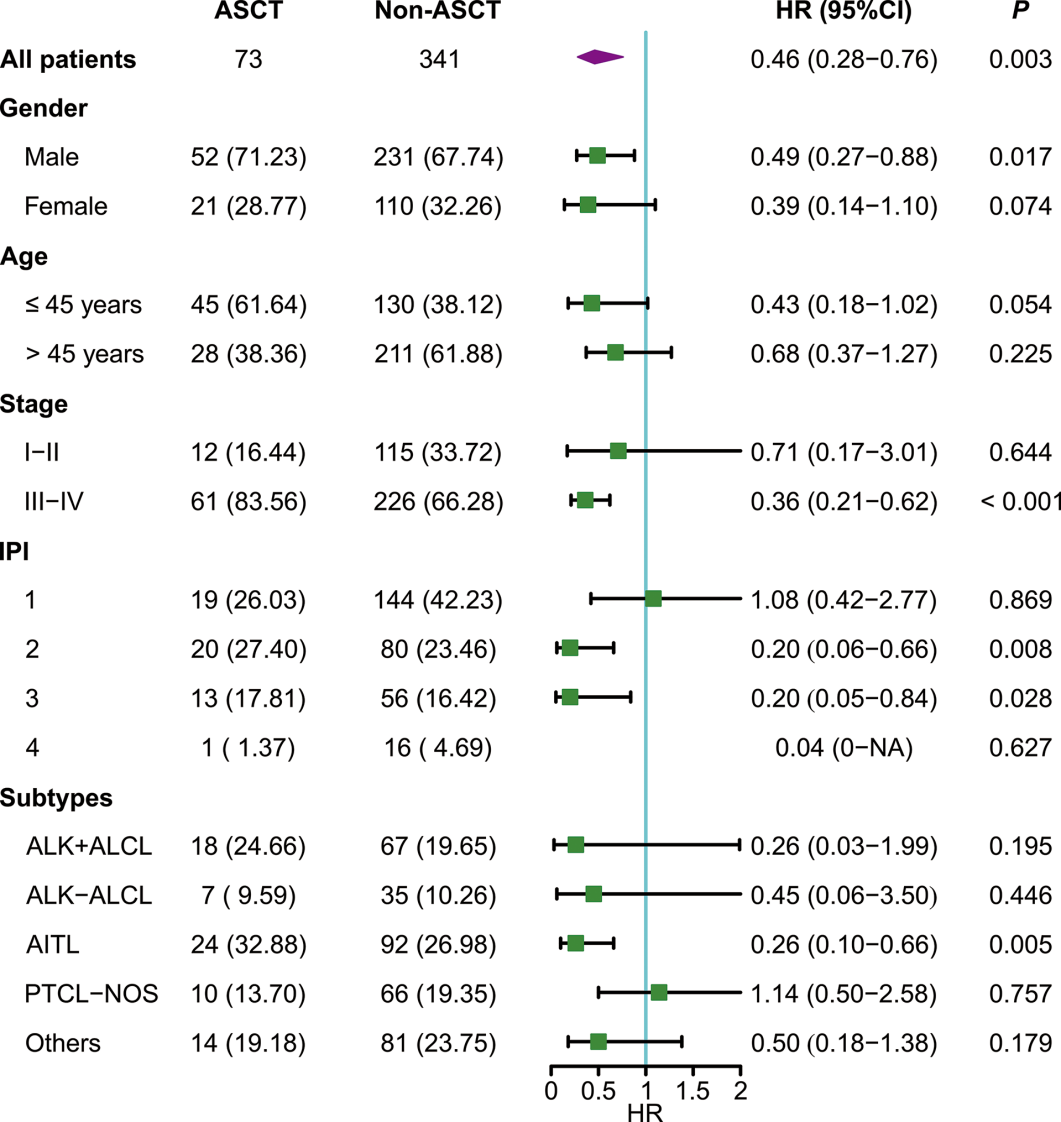
Subgroup analysis of event-free survival. ASCT, autologous stem cell transplantation; IPI, International Prognostic Index; AITL, angioimmunoblastic T-cell lymphoma; ALK+ALCL, anaplastic large-cell lymphoma, anaplastic lymphoma kinase positive; ALK-ALCL, anaplastic large-cell lymphoma, anaplastic lymphoma kinase negative; PTCL-NOS, PTCL not otherwise specified; HR, hazard ratio.

**Figure 3:**
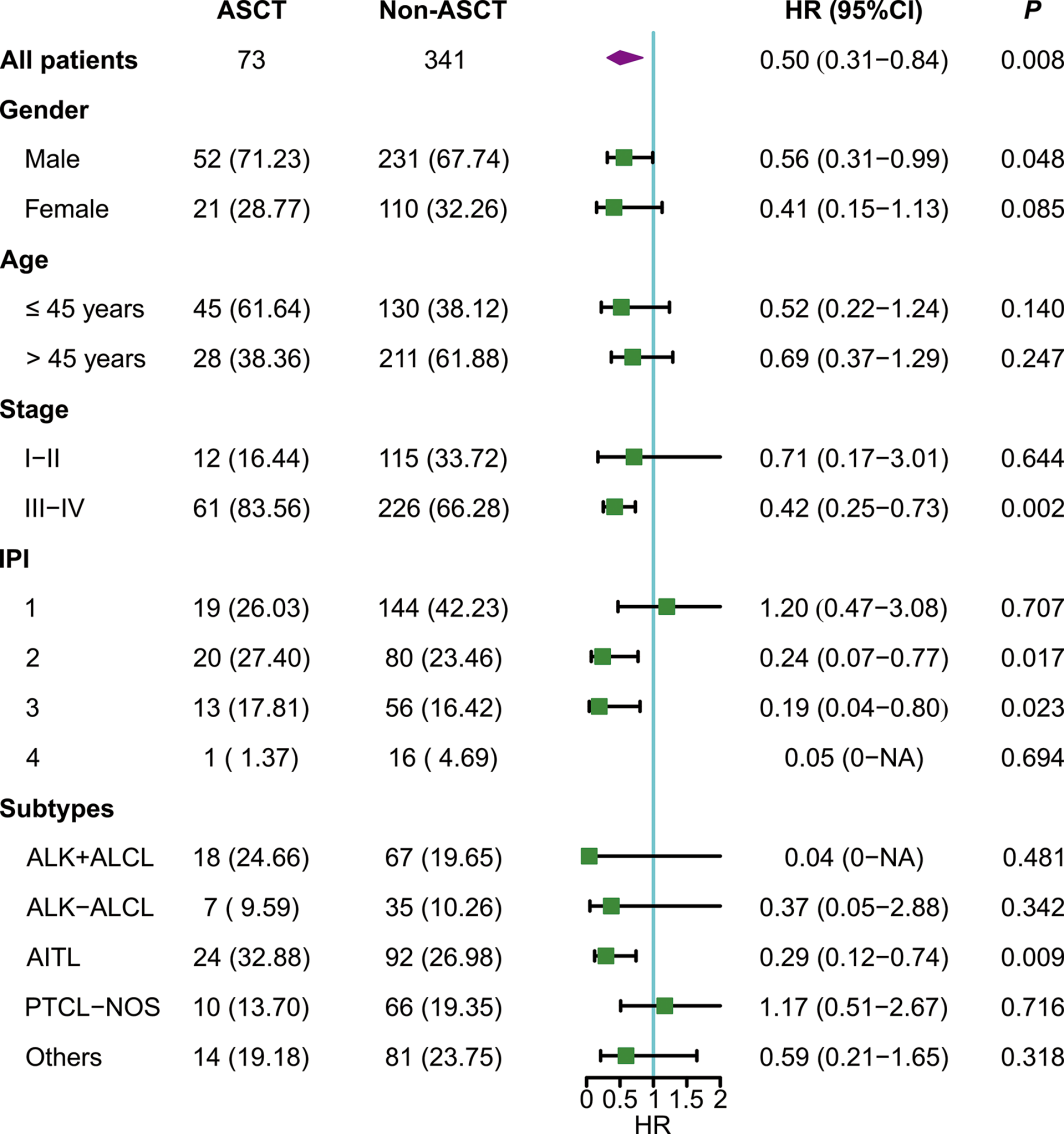
Subgroup analysis of overall survival.

For the whole cohort, ASCT was associated with better EFS (HR: 0.46 [95% CI: 0.28 - 0.76]) and OS (HR: 0.50 [95% CI: 0.31-0.84]) (**Figures 2**, **3**). Survival of patients with AITL was significantly better in the ASCT group than in the non-ASCT group (HR for EFS: 0.26 [95% CI: 0.10-0.66]; HR for OS: 0.29 [95% CI: 0.12-0.74]). However, there were no significant differences in EFS or OS between the ASCT and non-ASCT groups for ALK+ALCL, ALK-ALCL, AITL, PTCL-NOS, and other subtypes. Further subgroup analysis showed that ASCT was associated with better EFS and OS in advanced-stage disease and in low-intermediate and intermediate-high risk subgroups (**Figures 2**, **3**). In multivariate Cox regression analysis, ASCT was confirmed as the independent risk factors for OS in PTCL patients with CR1 or PR1 (**Table 2**).

**Table 2:** Univariate and multivariate Cox regression analysis for OS.

	Univariate Cox model		Multivariate Cox model	
	HR (95% CI)	*P*	HR (95% CI)	*P*
**Age**	1.034 (1.024-1.045)	< 0.001	1.024 (1.011 -1.037)	0.001
**Female**	0.988 (0.705-1.384)	0.944	–	–
**III–IV stage**	2.140 (1.429-3.204)	< 0.001	1.899 (1.194 -3.02)	0.014
**ASCT**	0.505 (0.305-0.836)	0.008	0.478 (0.242 -0.945)	0.034
**PR to the 1st treatment**	2.824 (2.060-3.873)	< 0.001	2.687 (1.859 -3.884)	< 0.001
**ALK+ALCL**	0.101 (0.041-0.245)	< 0.001	0.478 (0.242 -0.945)	0.003
**ECOG ≥2 (%)**	1.507 (0.851-2.668)	0.159	–	–
**LDH ≥ULN (%)**	1.531 (1.070-2.191)	0.020	1.138 (0.774 -1.673)	0.584
**B symptoms (%)**	1.112 (0.299-4.128)	0.875	–	–

ALK+ALCL, anaplastic large-cell lymphoma; anaplastic lymphoma kinase positive; ECOG, Eastern Cooperative Oncology Group performance status; LDH, lactate dehydrogenase; ULN, upper limit of normal; HR, hazard ratio; ASCT, autologous stem cell transplantation; PR, partial response.

## Discussion

PTCL has a poorer prognosis than aggressive B-cell lymphomas, with less durable remissions after anthracycline-containing multiagent chemotherapy (4, 28, 29). Treatment is particular challenging due to the relative rarity and biological heterogeneity of PTCL. The value of ASCT in PTCL remains controversial (9, 10, 22, 30), lacking a large sample retrospective study from China. The present study provides evidence in support of the use of ASCT in Chinese chemotherapy responders.

Previous studies suggest that consolidation with ASCT may not offer survival advantages for patients with PTCL except in specific clinical scenarios (10, 21, 22, 31). The COMPLETE study was the first large prospective cohort study to directly compare survival outcomes of patients with nodal PTCL in CR1 treated with or without consolidative ASCT. Eighty-three patients did not undergo ASCT, while 36 underwent consolidative ASCT. After a median follow-up of 2.8 years, no statistically significant difference was found in median OS between the ASCT group and the non-ASCT group (median OS: “not reached” vs. 57.6 months, respectively, *P* = 0.06). ASCT had no impact on the prognosis of PTCL-NOS but significantly improved OS and progression-free survival for patients with AITL; this finding is consistent with our results. Thus, it appears that patients with AITL are likely to benefit most from ASCT consolidation (16).

Our results were contrary to the findings of the large retrospective multicenter study based on the LYSA registry although both studies had similar designs. The LYSA study evaluated 269 patients with PTCL achieving CR1/PR1. The PTCL subtypes in the LYSA study included PTCL-NOS (29%), AITL (46%), and ALK-ALCL (25%). Among them, 134 patients were allocated to ASCT and 135 did not. Both Cox regression analysis and PSM analysis showed no survival advantage with ASCT consolidation (22). Our study enrolled more histologic subtypes (including ALK+ALCL, Enteropathy-associated T-cell lymphoma (EATL), and others); the differences between the study populations in the two studies may be one of the reasons for the contrary results. In addition, the performance of the intention-to-treat approach in this study may have led to more conservative estimates (32).

Although several earlier studies have reported that PTCL patients who receive upfront ASCT consolidation have a relatively good prognosis (5, 14, 15, 17, 33–35), none of the studies attempted to determine the sub-cohort of patients most likely to benefit. One pooled analysis showed that PTCL-NOS and ALK-ALCL patients without *DUSP22* rearrangement could benefit from ASCT (36), indicating ALK-ALCL patients with *DUSP22* rearrangement may be a potential transplant-exempt population. Thus, current evidence suggests clear benefits with ASCT consolidation in patients with AITL and uncertain benefits in patients with PTCL-NOS (probably due to the high heterogeneity in this subtype). The benefit of ASCT in different pathological subtypes needs further study.

Our results also demonstrated that upfront consolidative ASCT can improve prognosis in low-intermediate and intermediate-high risk subgroups of PTCL. Because only one patient in the high-risk subgroup underwent ASCT, we did not analyze the high-risk group. The evidence for the long-term efficacy of upfront ASCT in patients with early-stage and low-risk IPI is insufficient (21).

Because some previous studies have suggested that the frontline chemotherapy regimen may be associated with survival outcomes (37), we compared the survival difference between patients receiving CHOP vs. CHOPE as an initial regimen after matching baseline characteristics by PSM (data not shown). No survival difference was observed in our cohort.

In recent years, the discovery and clinical application of molecular biomarkers and signatures have provided unprecedented insights into PTCL (38–41). Novel antibody-drug conjugates and epigenetic modifying agents have shown promising clinical efficacy (42, 43). Of note, The ECHELON-2 study demonstrated a significant improvement in prognosis with frontline brentuximab vedotin (BV) plus cyclophosphamide, doxorubicin, and prednisone (A+CHP) compared with CHOP in CD30-positive PTCLs (43). Furthermore, Savage et al. recently conducted an exploratory subgroups analysis of ECHELON-2 trial supporting the role of consolidation consolidative stem cell transplant (autologous or allogeneic) in CD30-positive PTCL who achieve a CR following treatment with A+CHP. Patients who underwent stem cell transplants achieved a 64% reduction in the risk of a PFS event (HR: 0.36 [95% CI, 0.17-0.77]). The results indicate that even in the era of BV, consolidative stem cell transplant is still required (44).

Our results should be carefully interpreted because of some limitations in this study. First, this was a retrospective single-center study. The findings from the subgroup analysis may not be reliable because of the limited number of patients. Second, there is bound to be a discrepancy in response evaluation by PET/CT vs. CT (26). The CR rate may be underestimated for those patients who received response evaluation only by CT in the cohort. Finally, the systems of criteria for diagnosis have undergone changes in recent 20 years (1, 23–25). We enrolled patients according to the diagnostic criteria of that time, but there must be variability around the exact criteria used for diagnosis over long time span. Therefore, pathological review and molecular typing to define the subtypes of PTCL are required in future studies.

## Conclusion

ASCT consolidation may prolong survival in Chinese patients with PTCL showing complete or partial response to initial chemotherapy; benefit is most likely in patients with AITL. Patients with higher-risk IPI score and advanced-stage disease may also benefit significantly from ASCT. Our findings need to be confirmed in large controlled prospective studies.

## Data availability statement

The raw data supporting the conclusions of this article will be made available by the authors, without undue reservation.

## Ethics statement

The studies involving human participants were reviewed and approved by the ethics committee of Peking University Cancer Hospital. Written informed consent from the participants’ legal guardian/next of kin was not required to participate in this study in accordance with the national legislation and the institutional requirements.

## Author contributions

HG and WL designed this research. HG collected the data, performed the analysis and prepared the original draft. XJ was involved in data cleaning and analysis. MW and ND provided helpful suggestions for this study. All authors contributed to the article and approved the submitted version.

## Funding

This work was supported by the National Natural Science Foundation of China [grant numbers 82070205, 81870154]; the Clinical Research Fund For Distinguished Young Scholars of Beijing Cancer Hospital [grant number QNJJ202106]; and the Capital’s Funds for Health Improvement and Research [grant numbers 2022-1-2152, 2020-2Z-2157].

## Acknowledgments

We thank all the colleagues at Peking University Cancer Hospital & Institute for their support.

## Conflict of interest

The authors declare that the research was conducted in the absence of any commercial or financial relationships that could be construed as a potential conflict of interest.

## Publisher’s note

All claims expressed in this article are solely those of the authors and do not necessarily represent those of their affiliated organizations, or those of the publisher, the editors and the reviewers. Any product that may be evaluated in this article, or claim that may be made by its manufacturer, is not guaranteed or endorsed by the publisher.

## Glossary

**Table:**

AITL	angioimmunoblastic T-cell lymphoma
ALK+ALCL	anaplastic lymphoma kinase positive anaplastic large-cell lymphoma
ALK-ALCL	anaplastic lymphoma kinase negative anaplastic large-cell lymphoma
ASCT	autologous stem cell transplant
CHOP	cyclophosphamide, doxorubicin, vincristine and prednisolone
CHOPE	cyclophosphamide, doxorubicin, vincristine, prednisolone, etoposide
CI	confidence interval
CR1	first-time complete remission
ECOG	Eastern Cooperative Oncology Group
EFS	Event-free survival
HR	hazard ratio
IPI	International Prognostic Index
LYSA	Lymphoma Study Association
OS	overall survival
PET/CT	positron emission tomography/computed tomography
PR1	first-time partial remission
PSM	Propensity score matching;
PTCL	Peripheral T/NK-cell lymphoma
PTCL-NOS	peripheral T-cell lymphoma not otherwise specified
