# Supplementary material for: Effect of autologous hematopoietic stem cell transplantation for patients with peripheral T-cell lymphoma in China: A propensity score-matched analysis
Source: Front Oncol. 2022 Nov 17;12:1039888. doi: 10.3389/fonc.2022.1039888 (PMC9712948; doi:10.3389/fonc.2022.1039888)
Supplement: Supplementary file 1 [file DataSheet_1.docx]

**Supplementary Figure 1 Flowchart**


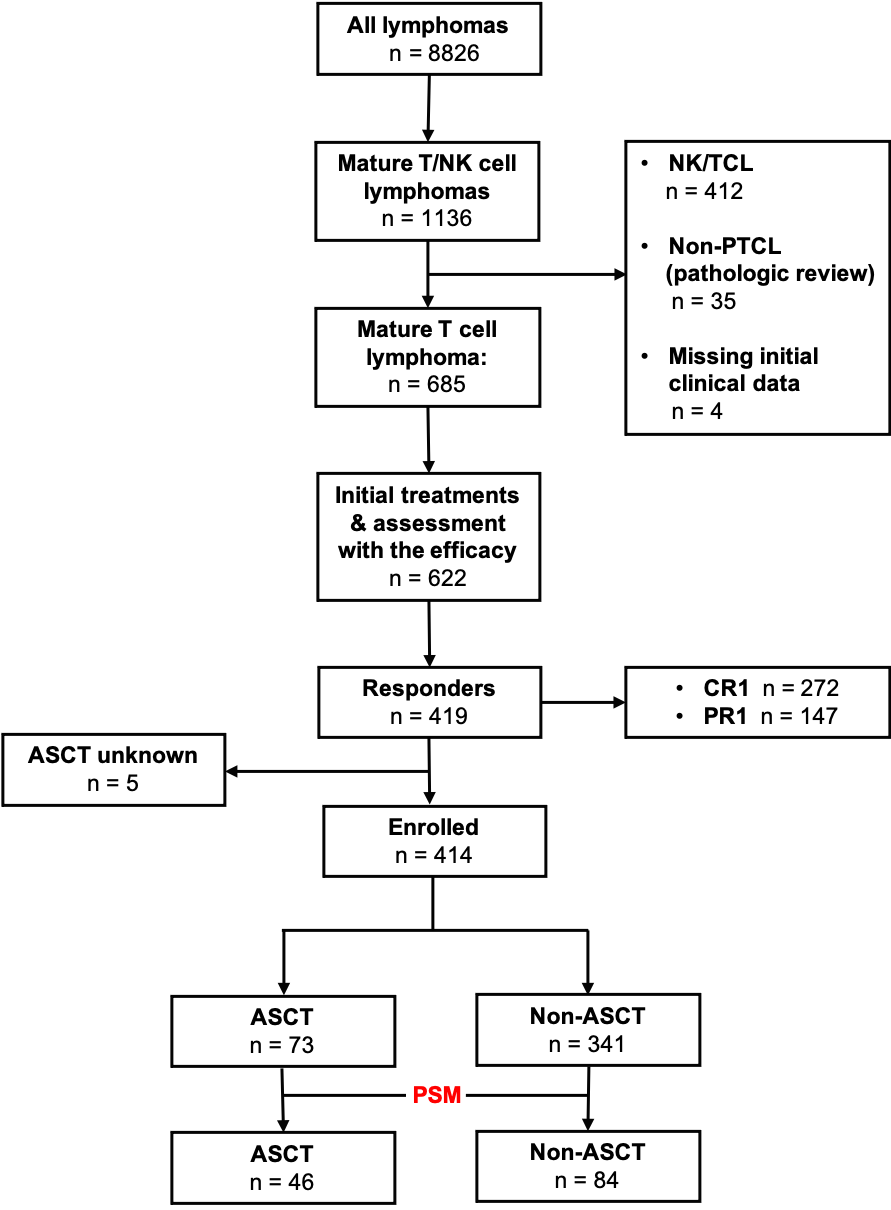


Propensity score matching was performed to adjust for differences in baseline characteristics between ASCT and non-ASCT groups, with the 1:2 matching described in the Statistical analysis section. However, due to the limitation of sample size, the preset matching degree cannot generally be fully achieved. We finally selected the present data to maximize the use of the clinical information.

NK/TCL: Natural killer/T cell lymphoma; ASCT: autologous stem cell transplantation; CR1: first complete remission; PR1: first partial remission

**Supplementary Figure 2 Subgroup analysis of event-free survival (EFS) and overall survival (OS)**

**
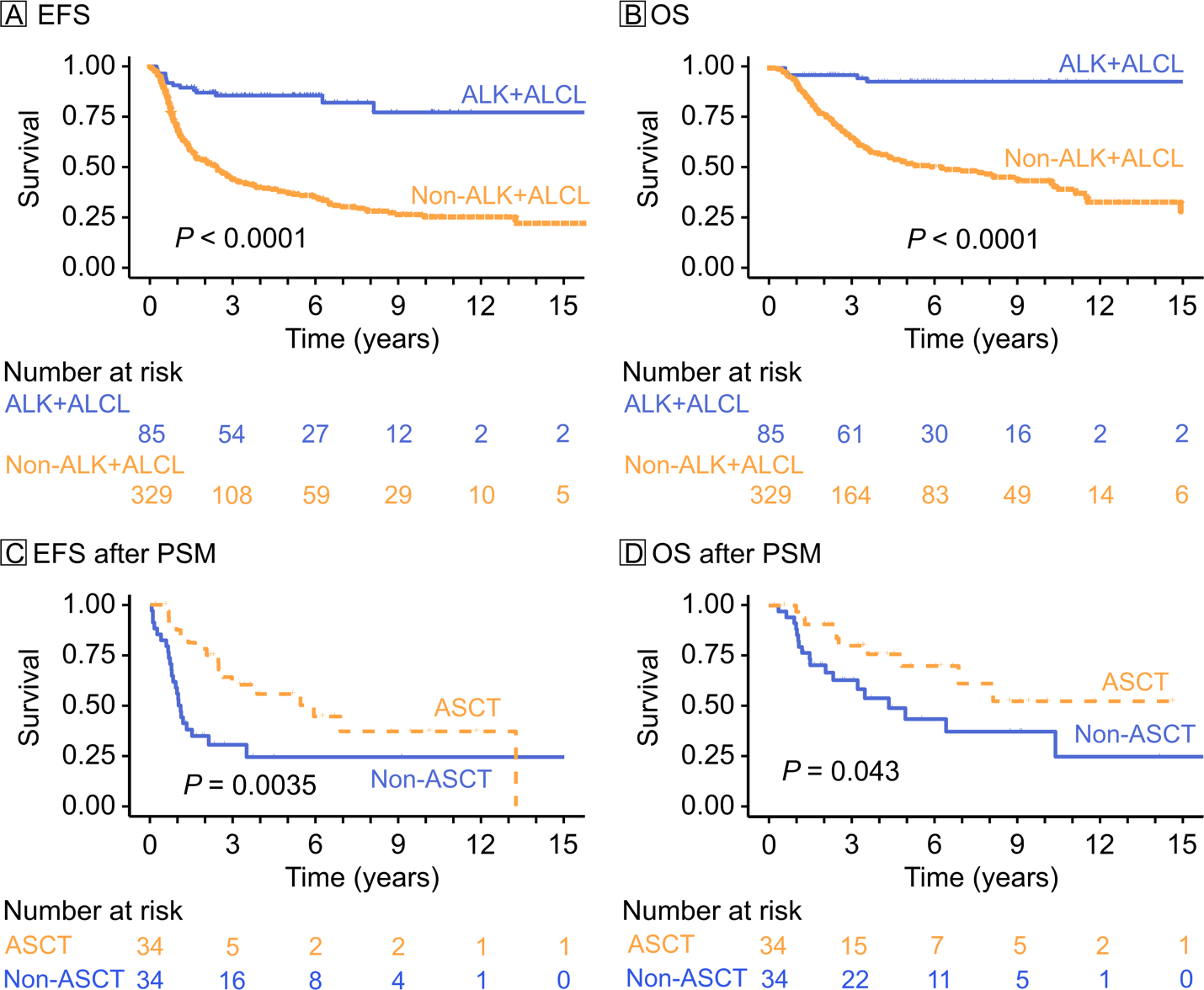
**

The EFS (A) and OS (B) were shown in ALK+ALCL vs. non-ALK+ALCL. For the Non-ALK+ALCL cohort, patients who underwent ASCT consolation had significantly favorable EFS (C) and OS (D) compared to the patients without ASCT after balancing the characteristics of patients (Propensity score matching [PSM] methods with a ratio of 1:1).

**Supplementary Figure 3** **EFS and OS in the CR group and the PR group**


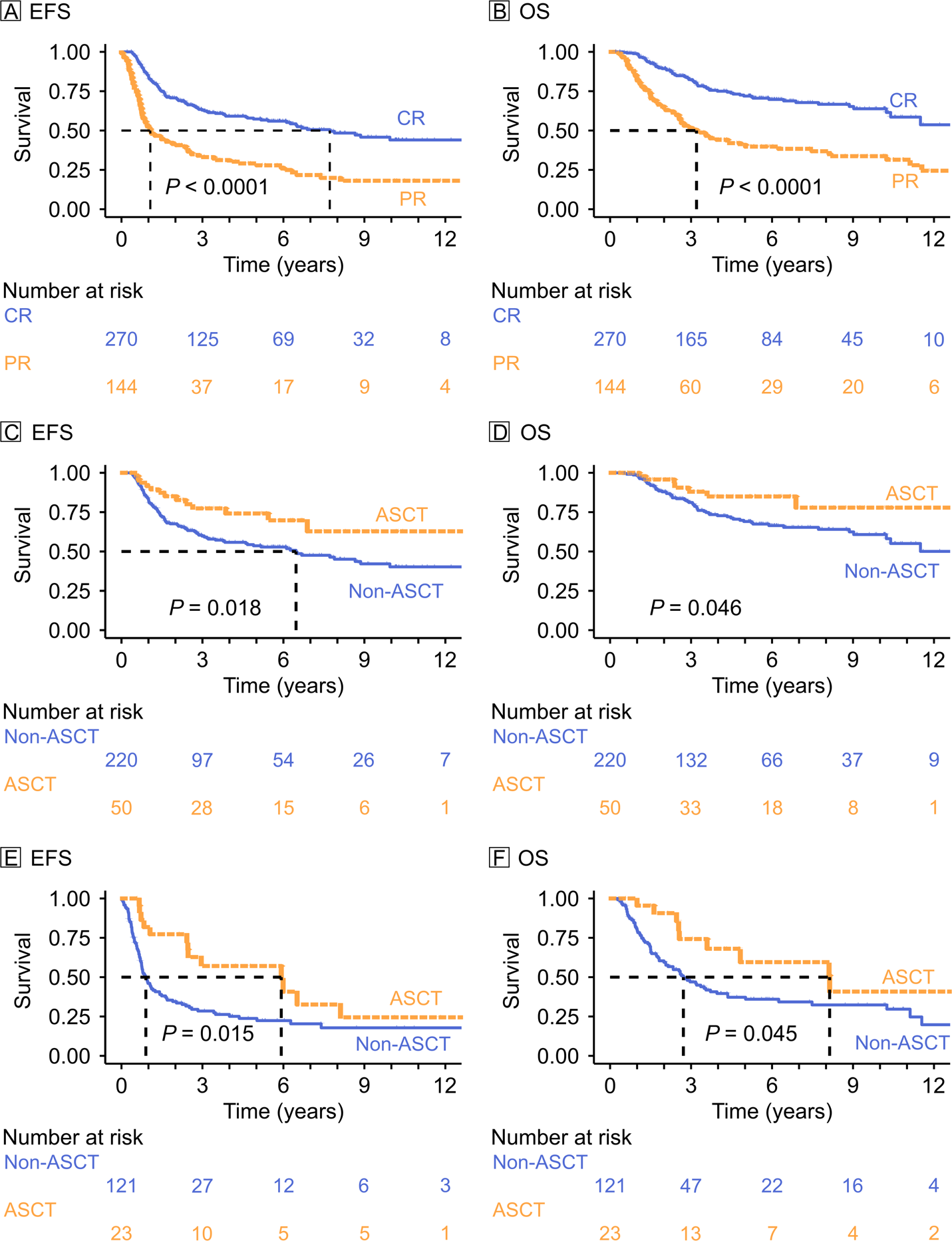


EFS: event-free survival; OS: overall survival; ASCT: autologous stem cell transplantation;

CR: complete remission; PR: partial remission

Comparison of EFS (A) and (B) between CR and PR patients based on the whole cohort (n=414); Survival analysis of EFS and OS in patients with CR (C, D)) and PR (E, F).

Meier analysis and the log-rank test was used to compare survival between groups.

**Supplementary Table 1 First-line regimens in the entire cohort**

|  | **AITL** | **ALK-ALCL** | **ALK+ALCL** | **PTCL-NOS** | **Others** | **Overall** |
| --- | --- | --- | --- | --- | --- | --- |
|  | n = 116 | n = 42 | n = 85 | n = 95 | n = 76 | n = 414 |
| **CHOP** | 55 (47.4) | 16 (38.1) | 21 (24.7) | 66 (69.5) | 26 (34.2) | 184 (44.4) |
| **CHOPE** | 6 (5.2) | 1 (2.4) | 4 (4.7) | 1 (1.1) | 15 (19.7) | 27 (6.5) |
| **CVP** | 11 (9.5) | 3 (7.1) | 1 (1.2) | 5 (5.3) | 6 (7.9) | 26 (6.3) |
| **CHOP-**  **EP/PEP** | 41 (35.3) | 18 (42.9) | 58 (68.2) | 10 (10.5) | 23 (30.3) | 150 (36.2) |
| **CHOP(E)/**  **GDP(Gemox)** | 0 (0.0) | 0 (0.0) | 0 (0.0) | 7 (7.4) | 1 (1.3) | 8 (1.9) |
| **Others** | 3 (2.6) | 4 (9.5) | 1 (1.2) | 6 (6.3) | 5 (6.6) | 19 (4.6) |

CHOP: cyclophosphamide, doxorubicin, vincristine, prednisolone; CHOPE: CHOP plus etoposide; CHOP-EP/PEP: CHOP plus etoposide and cisplatin; CVP: cyclophosphamide, vincristine prednisolone; CHOPE/GemOx: CHOPE alternating with gemcitabine and oxaliplatin; CHOPE/GDP (CHOPE alternating with gemcitabine, cisplatin, and dexamethasone); Other CT regimens: ESHAP (etoposide, methylprednisolone, cytosine arabinose, cisplatin); COMP (cyclophosphamide, vincristine, methotrexate, prednisolone); CHEP (cyclophosphamide, adriamycin, etoposide, prednisolone); GDP (gemcitabine, dexamethasone, cisplatin), SMILE (dexamethasone, methotrexate, ifosfamide, L-asparaginase, etoposide), and oral thalidomide; AITL: angioimmunoblastic T-cell lymphoma; ALK+ALCL: anaplastic large-cell lymphoma, anaplastic lymphoma kinase positive; ALK-ALCL: anaplastic large-cell lymphoma, anaplastic lymphoma kinase negative; PTCL-NOS: PTCL not otherwise specified

**Supplementary Table 2 Characteristics, response, and survival stratified by the initial treatment (CHOP or CHOPE regimen) in an expanded cohort (n = 532)**

|  | **CHOP** | **CHOPE** | ***P*** |
| --- | --- | --- | --- |
|  | **n = 307** | **n = 225** |  |
| **Age, years, median (IQR)** | 56.00 **(**37.50, 67.00**)** | 46.00 **(**29.00, 57.00**)** | **< 0.001** |
| **Female (%)** | 109 (35.5) | 67 (29.8) | 0.196 |
| **B symptoms** | 173 (56.4) | 103 (45.8) | **0.034** |
| **III–IV stage (%)** | 238 (77.5) | 160 (71.1) | 0.114 |
| **LDH ≥ULN (%)** | 112 (36.5) | 78 (34.7) | 0.842 |
| **ECOG ≥2 (%)** | 31 (10.1) | 9 (4.0) | **0.022** |
| **B symptoms (%)** | 38 (12.4) | 23 (10.2) | **0.046** |
| **Subtypes (%)** |  |  |  |
| ALK+ALCL | 25 (8.1) | 64 (28.4) | **< 0.001** |
| ALK-ALCL | 30 (9.8) | 25 (11.1) | 0.721 |
| PTCL-NOS | 48 (15.6) | 40 (17.8) | 0.5899 |
| AITL | 93 (30.3) | 66 (29.3) | 0.8862 |
| Others | 111 (36.2) | 30 (13.3) | **< 0.001** |
| **Respond to first treatment (%)** |  |  |  |
| CR | 103 (36.1) | 114 (54.3) | **< 0.001** |
| PR | 84 (29.5) | 37 (17.6) | **0.004** |
| **ASCT consolidation** | 24 (8.2) | 36 (16.5) | **0.006** |
| **Survival analysis** |  |  |  |
| 5-year EFS (95% CI) | 26.1% (95% CI: 21.3%-32.8%) | 43.2% (95% CI: 36.7%-50.9%) | **< 0.001** |
| 5-year OS (95% CI) | 41.3% (95% CI: 35.5%-48.2%) | 63.5% (95% CI: 56.4%-71.4%) | **< 0.001** |

IQR: interquartile range; ASCT: autologous stem cell transplantation;

Statistically significant associations are shown in bold (*P* < 0.05).

**Supplementary Table 3 Survival for ALK+ALCL vs Non-ALK+ALCL**

|  | **ALK+ALCL** | **Non-ALK+ALCL** | ***P*** |
| --- | --- | --- | --- |
|  | **n = 85** | **n = 414** |  |
| Median EFS, yeas (95% CI) | NR | 2.38 (1.64-2.99) | **< 0.001** |
| Median OS, yeas (95% CI) | NR | 6.28 (4.36-10.2) | **< 0.001** |
| 5-year EFS (95% CI) | 90.9% (84.6%-97.7%) | 37.5% (32.1%-43.7%) | **< 0.001** |
| 5-year OS (95% CI) | 93.2% (87.5%-99.2%) | 52.6% (46.9%-59.1%) | **< 0.001** |

IQR: interquartile range; ASCT: autologous stem cell transplantation; NR: Not reached.

Statistically significant associations are shown in bold (*P* < 0.05).

**Supplementary Table 4 Survival for Non-ALK+ALCL cohort after PSM**

|  | **ASCT** | **Non-ASCT** | ***P*** |
| --- | --- | --- | --- |
|  | **n = 34** | **n = 34** |  |
| Median EFS, yeas (95% CI) | 5.9 (2.6-NA) | 1.1 (0.8-3.5) | **0.004** |
| Median OS, yeas (95% CI) | NA | 4.4 (2.3-NA) | **0.043** |
| 5-year EFS (95% CI) | 55.7% (39.9%-77.6%) | 24.4% (12.2%-48.6%) | **< 0.001** |
| 5-year OS (95% CI) | 70.0% (53.9%-90.9%) | 43.6% (27.7%-68.6%) | **< 0.001** |

PSM: Propensity score matching
